# Mr. Chalmers, on Dr. Bellamy's Case of Diseased Bladder

**Published:** 1806-09

**Authors:** W. Chalmers

**Affiliations:** Surgeon. Shorncliff Camp


### *To the Editors of the Medical and Physical Journal.*

GENTLEMEN,

IN the character of one of the "*medical public,*" addressed by Dr. Bellamy, of his Majesty's ship Glory, in his publication of a most wonderful affection in perinæo, I beg leave, through the medium of your Journal, to make a few remarks on the statement of this naval practitioner.

Mr. Andrew Sharp, the unfortunate patient, Dr. B. informs us, came under his care on the 22d of January, 1806, for an affection in perinæo. This affection the Dr. states to have been some degree of enlargement, attended with considerable hardness and pain. We are not favoured with the most accurate anatomical description of the seat of the disease, nor its previous history from the time the patient first complained. It appears strange, that the medical gentleman should labour under any doubt, whether the tumour was chronic or acute.

On the 22d, he "*supposes*" it to be "*a chronic thickening, or enlargement,*" and that, "*probably,*" the prostate gland was diseased.

On the 23d, he admits that this "*chronic thickening was undoubtedly larger, more extensive,* and (although hardly perceptible to the eye, yesterday) *very apparent.* By way of nota bene, *he should not wonder to see* it the *foundation of a fistula.*

On the 24th, he is sorry that, notwithstanding his great exertions at an attempt to promote absorption, the *tumour increases very much:* he says, it is attended with a *degree* of *inflammation, great pain,* yet (wonderful to relate) "*no re-action of the circulation.*"

Give

Give me leave to ask Dr. Bellamy, whether inflammation ever existed in any degree without re-action and pain?

He says, "*looks and feels weak*;" I suppose he means the patient, and that he "*shall still persevere to repel,*" and that for fear of a fistula. He orders for this purpose, the mercurial ointment and fomentation to be repeated: a sure mean of repulsion, no doubt! The fertility of Dr. Bellamy's mind in this instance must be apparent to every medical practioner.

On the 25th, "the tumour was still large, but not *pointed*." He says, fomentations give most relief, and admits the formation of matter, by hoping it will burst internally. The Dr. thinks this would save an external wound, or fistula. A very learned conjecture! He seems surprised that his patient has had no rigors during suppuration.

Do rigors always attend suppuration, even of more extent than Mr. Sharp's tumour seems to have been?

On the 26th, the "abcess is said to have been as large as a fist." The patient has "*considerable fever, not great nor any re-action of heat.*"

What constitutes fever? Mr. Sharp, says the Doctor, is not aware of the seriousness of his case, because it has not been explained to him.

He adds, that he must now endeavour to promote suppuration, and keeps the "*patient rather low,*" and continues the warm applications. The abscess burst in the evening, a day or two sooner than expectation; he does not tell us of this till the 27th, and remains silent, whether he was present at this juncture, and what steps he took. On the 27th, the day after the bursting of the tumor, the appearances were certainly singular. I refer the reader to the original. The practitioner did little more, he says, *than cleanse the sore*, and *reduce the swelling*. 'Twas only to day he discontinued the ung. hydr. The Doctor's remarks on the 28th, are so unprofessional, that I pass them over in silence. On the 29th an operation took place! no arterial saltus attended the hemorrhage! As to the division of the rectum, he speaks like a man void of anatomical knowledge. On the 30th, after many windings and turns, a great discovery is made of a fistula in perinæo, properly so called. The patient's health is said in one place to be good, and constitution strong, but in the evening it is marked with contradiction; notwithstanding the singular appearances on the 27th, it was only at a late hour on the 28th, that bark was commenced. It is painful and tedious to overrun this mass of absurdity. The symptoms,

 treatment, treatment, and remarks, are of themselves so frivolous and absurd, and at the same time conveyed in such harsh language, that no man can read the case without pitying the writer. He displays in that trifling publication, a lameness too general in the navy.—I should be sorry to hurt the feelings of a whole class of men, by throwing out aspersions injurious to their character; but were I to relate in the most candid terms of equity and truth, what I have seen during my service in the royal navy, humanity would shrink at the tale. Yet there are some few in that service, whose zeal for the character they bear, and welfare of our brave seamen, has stimulated them to exertions highly honourable, namely, to know their duty, and to do it.

I am, &c.

W. CHALMERS, A. M. *Surgeon.*

*Shorncliff Camp,*

*August 13th,* 1806.

